# Role of Steroids in Post-streptococcal Glomerulonephritis Without Crescents on Renal Biopsy

**DOI:** 10.7759/cureus.3150

**Published:** 2018-08-15

**Authors:** Tsu Jung Yang, Harshil Shah, Akindele Olagunju, Matthew Novak, William Difilippo

**Affiliations:** 1 Internal Medicine, Guthrie Clinic/Robert Packer Hospital, Sayre, USA; 2 Internal Medicine, Guthrie Clinic, Sayre, USA; 3 Department of Medicine, Geisinger Commonwealth School of Medicine/Robert Packer Hospital, Rochester, USA; 4 Nephrology, Guthrie Clinic/Robert Packer Hospital, Sayre, USA

**Keywords:** post streptococcal glomerulonephritis (psgn), steroids, crescents

## Abstract

Steroid is usually indicated in patients with post-streptococcal glomerulonephritis (PSGN) with more than 30% crescents on renal biopsy. The role of steroids in patients without crescentic glomerulonephritis is not clear. We present a 19-year-old male patient who was diagnosed with PSGN three weeks after a sore throat infection. He developed acute renal and respiratory failure requiring hemodialysis and mechanical ventilation. The renal biopsy confirmed PSGN, but did not show severe histological features such as crescents formation. Due to lack of clinical improvement, trials of pulse dose methylprednisolone were initiated with prompt improvement in renal and respiratory function. Our case suggested the potential role of high dose steroids in select patients of PSGN with progressive renal failure, development of multi-organ system deterioration, and failed conservative management irrespective of histological findings.

## Introduction

Acute post-streptococcal glomerulonephritis (PSGN), first described as a complication of scarlet fever in 18th century, is the prototype of post-infectious glomerulonephritis. It is an immune complex-mediated disease that is usually associated with recent streptococcal infection of skin or throat. While the annual incidence in developing countries is nine cases per 100,000 inhabitants [1], the incidence in developed countries has been declining. It is estimated to be 0.3 cases per 100,000 persons-year [2]. The typical clinical features vary from asymptomatic microscopic hematuria to acute nephritic syndrome (gross hematuria, proteinuria, edema, hypertension, and elevated serum creatinine). The prognosis is excellent especially in children with recovery within one week in a typical case. The requirement of chronic hemodialysis is rare [1]. However, some patients can develop rapidly progressive glomerulonephritis associated with crescents formation on renal biopsy. The current practice is to consider pulse corticosteroids in these particular cases although it is not proven to be beneficial [3]. However, the role of steroids in patients with progressive renal failure without severe histological features has not been studied to our knowledge.

## Case presentation

**Figure 1:**
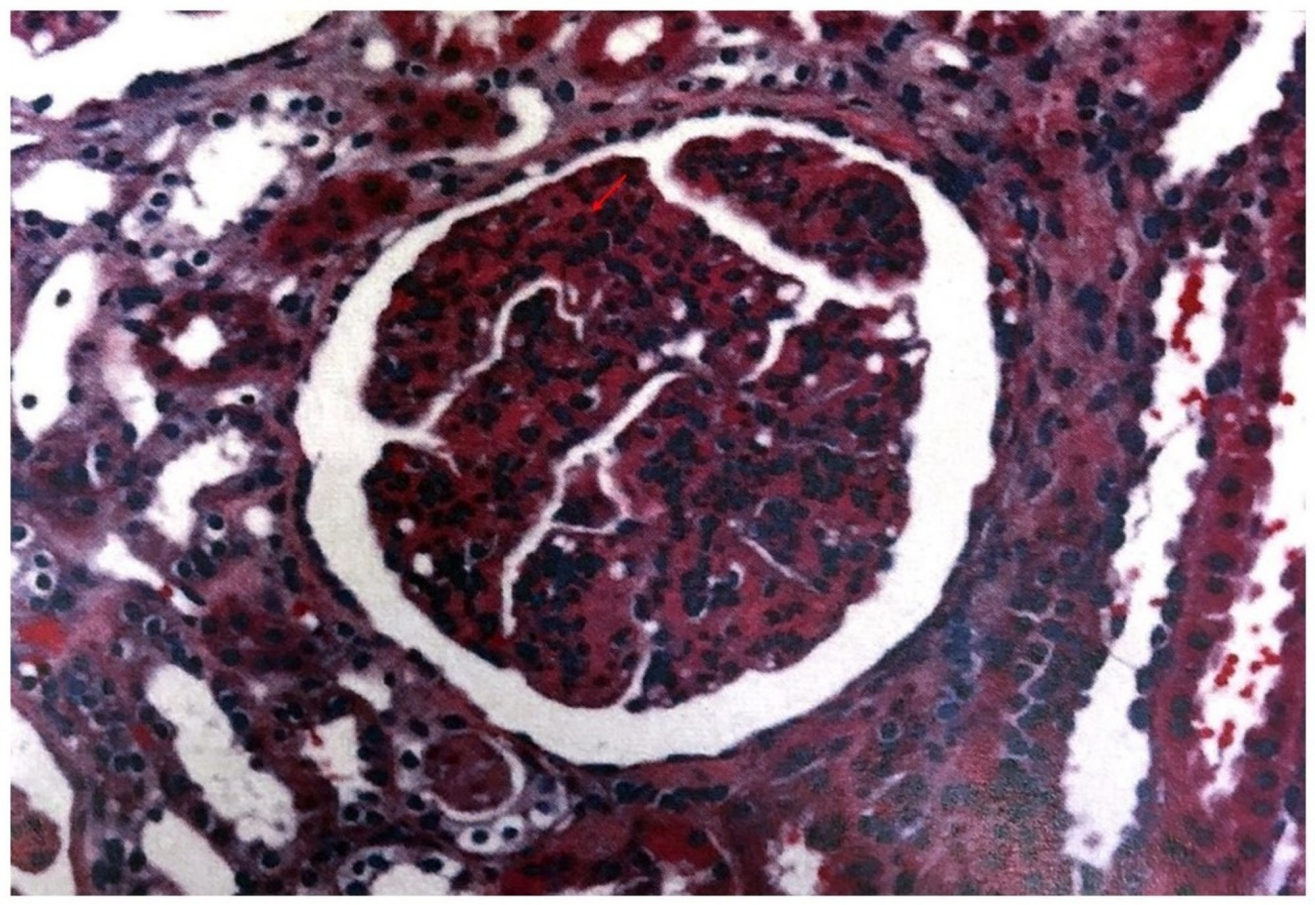
Light microscopy: the arrow shows endocapillary proliferation with cellular infiltrates.

**Figure 2:**
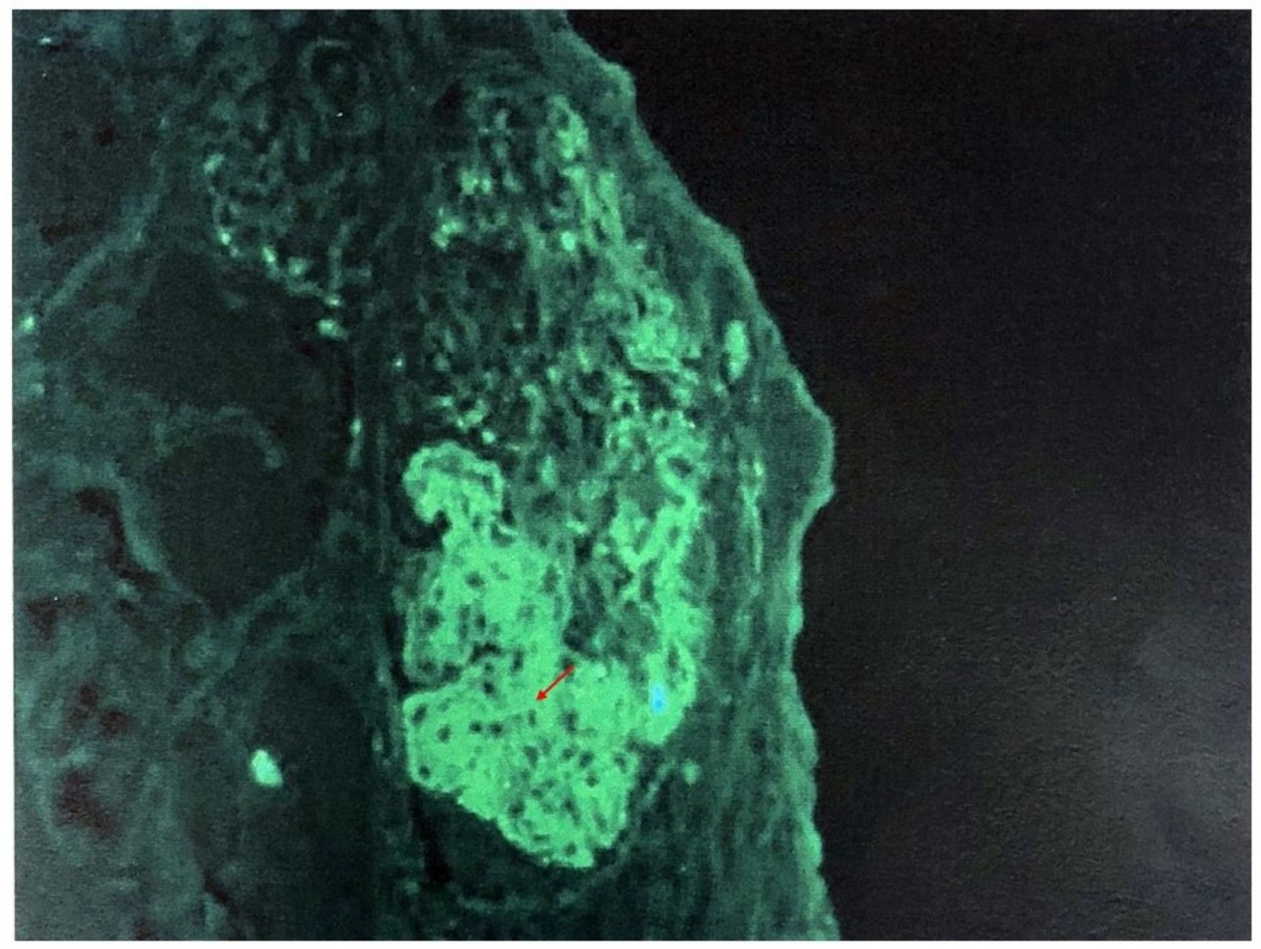
Direct immunofluorescence: the arrow shows granular staining of C3 complements.

**Figure 3:**
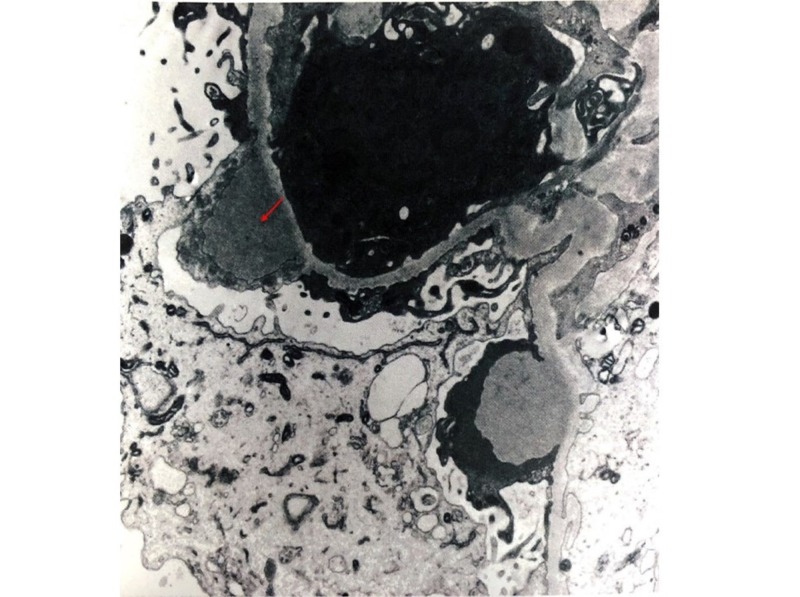
Electron microscopy: the arrow shows subepithelial hump-like immune deposits.

**Figure 4:**
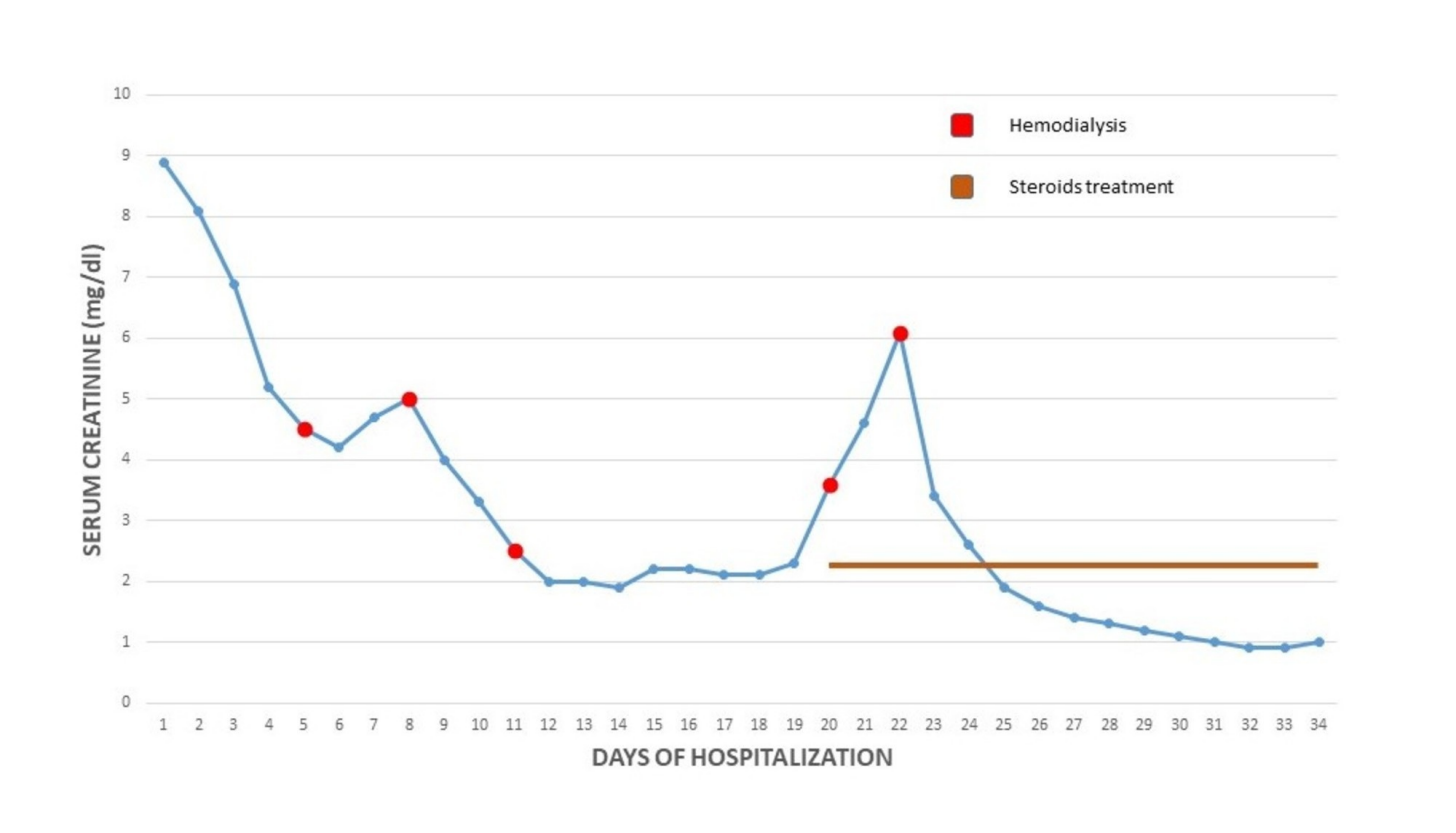
The trend of serum creatinine in relation to hemodialysis and steroids therapy.

We present a case of 19-year-old male patient with no significant past medical history who presented with cough, greenish sputum, severe nausea, vomiting, and diarrhea for four days. It was associated with subconjunctival hemorrhage which prompted him to seek for emergent care. The patient reported sore throat for three weeks prior which was treated with over-the-counter cold medications. Initial vitals reported a temperature of 98.6°F, heart rate 82 beats per minute, blood pressure 156/85 mmHg, and respiratory rate 20 per minute. On physical exam, the oropharynx did not show erythema or exudates, no palpable lymphadenopathy. Chest and abdominal exams were benign. Labs showed white cell count 12,800/uL, hemoglobin 14.1 g/dL, and platelet 154,000/uL, sodium 133 mmol/L, potassium 4.5 mmol/L, chloride 97 mmol/L, bicarb 19 mmol/L, blood urea nitrogen (BUN) 95 mg/dL, creatinine 8.9 mg/dL, calcium 9.1 mg/dL, and liver function tests were normal. Urine analysis showed amber color urine, specific gravity >1.030, pH 5, protein >300 mg/dL, negative glucose, large blood, trace ketones, moderate bilirubin, negative nitrite, negative leukocytes, white blood cell (WBC) 10–25/HPF, red blood cell (RBC) 10–25/HPF, hyaline cast 10–25/LPF, and granular cast 0–2/LPF. Estimated 24-hour urinary protein excretion was 0.6 g/day. He was admitted and given volume resuscitation and broadly covered with antibiotics by his primary service. Nephrology was consulted in view of acute renal failure, proteinuria, and hematuria. Initial differential diagnoses of his acute kidney injury included PSGN, severe dehydration, IgA nephropathy, and vasculitis. Rapid strep A screening and throat swab culture were negative. C3 and C4 complements were <40 and <8 mg/dL, respectively. Total complement level was <10 U/mL. Anti-DNASE B antibody titer was 770 U/mL, and anti-streptolysin O titer was 285 IU/mL. Autoimmune workup was negative except antinuclear antibody titer of 1:160, and positive cryoglobulin with low cryoprecipitate. Computed tomography (CT) abdomen and pelvis without contrast showed small bilateral pleural effusions, no renal masses or obstruction as well as normal appearing ureters and bladder. With supportive measures, creatinine improved initially, but blood urea nitrogen got worse. On day three of admission, he developed pulmonary congestion and diuresis was tried without success. He subsequently developed uremic symptoms. Intermittent hemodialysis was started as supportive therapy for PSGN, volume overload, and uremic symptoms. In spite of aggressive conservative therapy, he continued to be hypoxemic with persistent bilateral pulmonary infiltrates. It was suspected that he had sequelae of pulmonary-renal syndrome despite negative serology. Thus, he underwent bronchoscopy and bronchoalveolar lavage which ruled out alveolar hemorrhage. He also underwent renal biopsy on day 22 of hospitalization. Renal biopsy identified acute tubular injury, enlarged glomeruli, endocapillary proliferation, neutrophils on light microscopy (Figure 1), granular staining in capillary loops for C3 on direct immunofluorescence (Figure 2), and subepithelial hump-like immune deposits on electron microscopy (Figure 3). He had no evidence of tubular atrophy or interstitial fibrosis. The biopsy findings were consistent with PSGN. He was intubated for acute hypoxemic respiratory failure, and intravenous pulse dose steroids were initiated. He received three days of 1 g intravenous methylprednisolone followed by 500 mg daily for six days. The serum creatinine rapidly came down with daily improvement in urine output without further dialysis requirements. The trend of serum creatinine in relation to hemodialysis and steroids therapy is demonstrated in Figure 4. He was discharged with 20 days tapering course of oral prednisone. On the day of discharge, his serum creatinine was 1 mg/dL. On follow-up, renal function remained to be normal. He no longer required supplemental oxygen, and prednisone was tapered off. The degree of proteinuria and hypertension also improved significantly.

## Discussion

PSGN is a disease that is mainly attributed by depositing immune complexes in the glomeruli which lead to local activation of immunoglobulins and complements. Two most recently studied antigens that claim to be responsible for nephritogenicity are nephritis-associated plasmin receptor (NAPlr), and streptococcal pyrogenic exotoxin B (SPEB) and its zymogen precursor (zSPEB) [1,4]. Anti-NAPlr antibody has been detected in the convalescent sera of PSGN patients [5]. SPEB antigenic deposits were demonstrated in the characteristic electron dense subepithelial deposits (humps) of PSGN [6-7]. These antigens can trigger antibody formation, and subsequently activate complement system. The alternative pathway is usually activated in PSGN, and it clinically manifests as depression of C3 level. However, C1 and C4 levels may be low in some patients which suggest the involvement of classic complement pathway. Autoimmune reactivity such as anti-IgG rheumatoid factors, anti-DNA antibodies, anti-C1Q antibodies, and anti-neutrophil cytoplasmic autoantibodies (ANCAs) has also been described in PSGN patients. The clinical relevance of this phenomenon remains uncertain [1]. The treatment of PSGN is usually supportive with excellent prognosis. It mainly focuses on management of hypertension, and edema. The use of immunosuppressive therapy in PSGN has been controversial [3,8]. Several studies have suggested the benefit of steroids in selected patients, but the exact mechanism is not clear [9-10]. The current expert consensus is to use steroids in patients with more than 30% crescents formation on renal biopsy. But, the role of steroids in patients without crescentic glomerulonephritis has not been studied. In the present case, the renal biopsy did not show any crescent formation, however, the patient did have evidence of significant endocapillary proliferation. It is postulated that the degree of antigen load, and subsequent antibody formation, may predate the degree to which the immune response creates renal injury. Thus, we suspected this may be causative to the ongoing renal deterioration our patient developed. Persistently low complement levels suggested continuous immune response activity in the present case. It is noted that complements may take up to six months to normalize. With the deterioration of respiratory function and ongoing dialysis requirements, we began pulse dose steroids for three days, followed by a high dose prednisone taper. Respiratory and renal function improved promptly with no further need for supplemental oxygen and normalization of serum creatinine at 1 mg/dL within 13 days of starting steroids. This suggested the role of immunosuppression with high dose steroids in select patients with progressive renal failure irrespective of histological findings or the development of multi-organ system deterioration.

## Conclusions

Acute PSGN is an immune-mediated disease with favorable prognosis but can develop progressive renal failure requiring hemodialysis. High dose systemic steroids have previously held a limited role in the management of this disease. However, this case demonstrated the benefit of high dose steroid immune modulation in a 19-year-old with proliferative, though not crescentic, renal disease and respiratory failure. Further studies need to be done to evaluate the routine use of steroids in these select cases of PSGN.
